# A two-year indoor–outdoor monitoring study of household clean air interventions during ambient and wildfire smoke periods in King County, Washington

**DOI:** 10.1088/2515-7620/ae69a6

**Published:** 2026-05-14

**Authors:** Katharine A Teigen, Mariana Cortes Espinosa, Hannah McKinley, Anna Reed, Elizabeth Berrang, Julio Ramos-Vazquez, C Addison Houston, Tania Busch Isaksen

**Affiliations:** 1 Department of Environmental and Occupational Health Sciences, University of Washington, Seattle, WA, United States of America; 2 Climate and Health Equity Initiative, Public Health—Seattle and King County, Seattle, WA, United States of America; 3 Evans School of Public Policy and Governance, University of Washington, Seattle, WA, United States of America; 4 Department of Health Systems and Population Health, University of Washington, Seattle, WA, United States of America; 5 Department of Epidemiology, University of Washington, Seattle, WA, United States of America

**Keywords:** air pollution, indoor, particulate matter, wildfires, climate change, home environment, Washington

## Abstract

Wildfire smoke (WFS) season is lengthening and intensifying with climate change, creating the need for effective adaptation strategies for reducing exposure to poor air quality during smoke events. To better understand the utility of low-cost air sensors and portable air filtration devices as household-level interventions for improving indoor air quality (IAQ) during WFS events, we launched a participatory science study among select King County, Washington households during summer 2023 and 2024, coinciding with wildfire season. Participants received indoor PurpleAir sensors, do-it-yourself (DIY) box fan filter kits, high efficiency particulate air (HEPA) portable air cleaners, and surveys on device use. We compared hourly fine particulate matter (PM_2.5_) concentrations from the indoor sensors to those from proximate outdoor PurpleAir sensors. We ran linear mixed-effects models to assess the influence of WFS and building characteristics on IAQ and to evaluate the efficacy of air filtration use (2024 only). Twelve households in 2023 and 24 households in 2024 completed the study. In 2023, the mean indoor and outdoor PM_2.5_ concentrations were higher than those in 2024 (a difference of 2.62 *µ*g m^−3^ and 1.74 *µ*g m^−3^, respectively), reflecting different trends in air quality and WFS events. In 2024, both filtration devices were associated with reductions in indoor PM_2.5_ concentrations; however, effect estimates differed after accounting for outdoor PM_2.5_, with HEPA portable air cleaners associated with a greater reduction than DIY box fan filters. The occurrence of a WFS event, warmer outdoor temperature, and higher appraised household value were significantly associated with higher indoor PM_2.5_ concentrations, although a large portion of the model’s variance remained unexplained. These findings suggest that low-cost air sensors and air filtration devices may be effective IAQ interventions and encourage future investigations with larger sample sizes and more precise measures of air filtration use.

## 1. Introduction

Driven in part by climate change, wildfires are increasing in frequency and intensity across the globe [1]. The burning of fossil fuels strengthens the greenhouse effect, raising temperatures and reducing snowpack, which dries out vegetation earlier in the season and creates ideal conditions for large-scale wildfires [2, 3]. In turn, these wildfires release heat-trapping gases, further accelerating climate change in a dangerous positive feedback loop [4]. While such large-scale wildfires are occurring globally, areas such as the western United States (US) are experiencing them at higher frequencies and intensities resulting in regional impacts to air quality from the smoke they produce [5]. Notably, the Pacific Northwest region of the US experienced some of the most severe wildfire events—defined by area burned, number of reburns and pollution generated—from 2006 to 2020 [6, 7].

Wildfire smoke (WFS) contains a unique mixture of pollutants and substantially worsens air quality in impacted areas. The exact composition of WFS depends on the type and quantity of fuels that are burned (i.e. natural versus anthropic, or forests versus grasslands), but common WFS pollutants include nitrogen dioxide (NO_2_), ozone (O_3_) precursors, polycyclic aromatic hydrocarbons (PAHs), lead, carbon dioxide (CO_2_), volatile organic compounds (VOCs), and particulate matter [8]. Of particular health concern during WFS events is exposure to fine particulate matter (PM_2.5_), or particulates with an aerodynamic diameter less than 2.5 *µ*m [9]. The exact composition of PM_2.5_ varies widely between combustion sources, but it is typically generated from the combustion of fossil fuels and vegetation, which makes PM_2.5_ a major component of WFS [10].

Inhaling WFS PM_2.5_ poses significant health threats, potentially related to its pattern of more extreme and infrequent concentrations, higher absorption into the body, and relatively higher elemental and organic carbon content compared to non-WFS PM_2.5_ [11]. Indeed, one study found that WFS PM_2.5_ may be up to 10 times more harmful to health than PM_2.5_ from other sources [12]. PM_2.5_ particles can enter the bloodstream, leading to a combination of short-term respiratory irritation and long-term impacts including cardiovascular disease, cognitive decline, and other chronic conditions [13–20]. While less research has been conducted on the health impacts of WFS-specific PM_2.5_, studies consistently identify elevated morbidity and mortality risks associated with short-and long-term exposures [21–23]. Indeed, an investigation during the 2020 wildfire season in Washington State found excess all-cause and respiratory mortality cases attributable to the increased WFS PM_2.5_ [23]. Under high emission scenarios, climate-related increases in WFS PM_2.5_ are projected to cause hundreds of thousands of additional asthma cases and an estimated $43 billion in annual health-related economic losses by the end of the century [24, 25].

During WFS events, public health guidance typically advises individuals to limit exposure to smoke and to remain indoors [26]. Given that the average American spends upwards of 90% of their time indoors, it is necessary to consider how indoor air quality (IAQ) is impacted during WFS events, as well as the associated health effects of common IAQ pollutants [27]. Indoor air may be up to 2–5 times more polluted than outdoor air, with many common household practices and activities contributing to this, such as using household cleaning products, cooking foods at high temperatures without proper ventilation, sweeping or vacuuming, and burning incense or candles [28].

Additionally, various building characteristics can significantly impact the quantity and type of pollutants that are able to enter households from the outside environment. Specifically, older buildings tend to be ‘leakier’, resulting in higher infiltration rates of outdoor air into indoor environments, typically through cracks or gaps in the building exterior [29]. Certain building construction types, such as wood-framed structures and those without weatherstripping around windows and doorways, are also associated with a substantially higher infiltration rate [30, 31]. Outdoor air pollutants can enter buildings through natural ventilation (i.e. the opening of windows and doors) and mechanical ventilation (i.e. heating, ventilation, and air conditioning systems and exhaust fans). To maintain IAQ and energy efficiency in buildings, balanced ventilation systems equipped with filtration media with a minimum efficiency rating value (MERV) of 13 or higher, which continuously replace stale indoor air with fresh, filtered outdoor air, can be employed [32]. However, mechanical ventilation systems commonly found in many residential buildings may not adequately filter outdoor air, especially when outdoor air pollutants are significant [33].

Low-cost air quality sensors can be used as a household-level intervention for the detection and reduction of indoor air pollution. Outdoors, they have served as a public health intervention in various urban settings to measure exposure to ambient urban air pollution, helping to guide the development of policies and regulations on source control and reduction [34]. Increasingly, low-cost sensors have been used to better understand the infiltration of WFS indoors during wildfire events, generally detecting higher indoor PM_2.5_ concentrations during WFS events or when air filtration devices were not used, compared to ambient or non-filtration use periods [35–37]. Notably, much of this work has been conducted by research teams in the Pacific Northwest and British Columbia, highlighting the region’s WFS vulnerability and need for affordable community-level interventions to promote WFS exposure reduction. Indeed, these sensors can provide hyper-localized spatiotemporal resolution of air pollutant concentrations that can be used to inform pollutant-management decisions. At the household and individual level, they provide building occupants with an improved awareness of their IAQ. This awareness can motivate behavioral changes and empower individuals’ exposure reduction actions such as using vent fans, opening windows, or operating portable air cleaners to reduce indoor particulate concentrations.

Increasing public awareness of IAQ and its effects on health have contributed to a rise in commercially available portable air cleaners, as well as increased popularity of do-it-yourself (DIY) air filtration devices. Portable air cleaners differ from a building’s central mechanical ventilation system, as they are portable, stand-alone units that are designed to reduce air pollution for single rooms or specific areas within a building [38]. To achieve this, many units utilize fibrous media air filters, such as high efficiency particulate air (HEPA) filters, for particle capture, coupled with a fan to circulate air through the filter media. Efficiency is measured via a clean air delivery rate in cubic feet per minute [38].

The US environmental protection agency (EPA) has identified numerous studies on portable air cleaners that have shown statistically significant reductions in particles including PM_2.5_ and coarse particulate matter (PM_10_) [38]. In a meta-analysis of 148 field studies, authors reported that the use of portable air cleaners were generally effective at removing PM_2.5_ and PM_10_, with average reductions of 49% and 44% respectively [33]. These studies primarily focused on residential building settings and portable air cleaners with HEPA filters or activated carbon [33]. Only eight of the studies included in the meta-analysis investigated WFS as the pollutant type. This exemplifies the need for additional studies on the efficacy of portable air cleaners for the removal of wildfire-related particulate matter in indoor settings.

This pilot study leveraged participatory citizen science to evaluate the interplay of building characteristics, indoor and outdoor PM_2.5_ concentrations, and behavioral adaptations during the summers of 2023 (17th May–31st August) and 2024 (8th July–13th September). These periods coincided with a few WFS events in King County, WA. Specifically, there were two WFS events in 2023 (5th July and 21–29th August) and one in 2024 (5–8th September). The 5 July 2023 event was the result of a combination of 4th of July fireworks and WFS from British Columbia, with air quality in King County reaching ‘unhealthy for sensitive groups’ [39]. Air quality levels reached ‘unhealthy’ levels during 21–29th August, 2023 and ‘moderate’ levels during 5–8th September, 2024 [40, 41]. This study was implemented following three consecutive years of particularly severe WFS seasons in King County, occurring in 2020, 2021, and 2022 [42–44]. Thus, a better understanding of WFS exposures at the household-level and the utility of low-cost, health protective interventions were primary motivators of this study. Our research questions were:

(1) How do PM_2.5_ concentrations, as measured by PurpleAir sensors, differ between indoor and outdoor contexts across the two study years?
(2) How effective are DIY box fan filter kits and HEPA portable air cleaners at reducing indoor PM_2.5_ concentrations?
(3) How do WFS and building characteristics influence indoor PM_2.5_ concentrations?

The findings of this community centered two-year pilot study aim to increase understanding of how IAQ monitoring, the use of air filtration devices, and related risk communication can work symbiotically to safeguard public health in residential environments by improving IAQ.

## 2. Methods

### 2.1. Study overview

**Figure 1.**
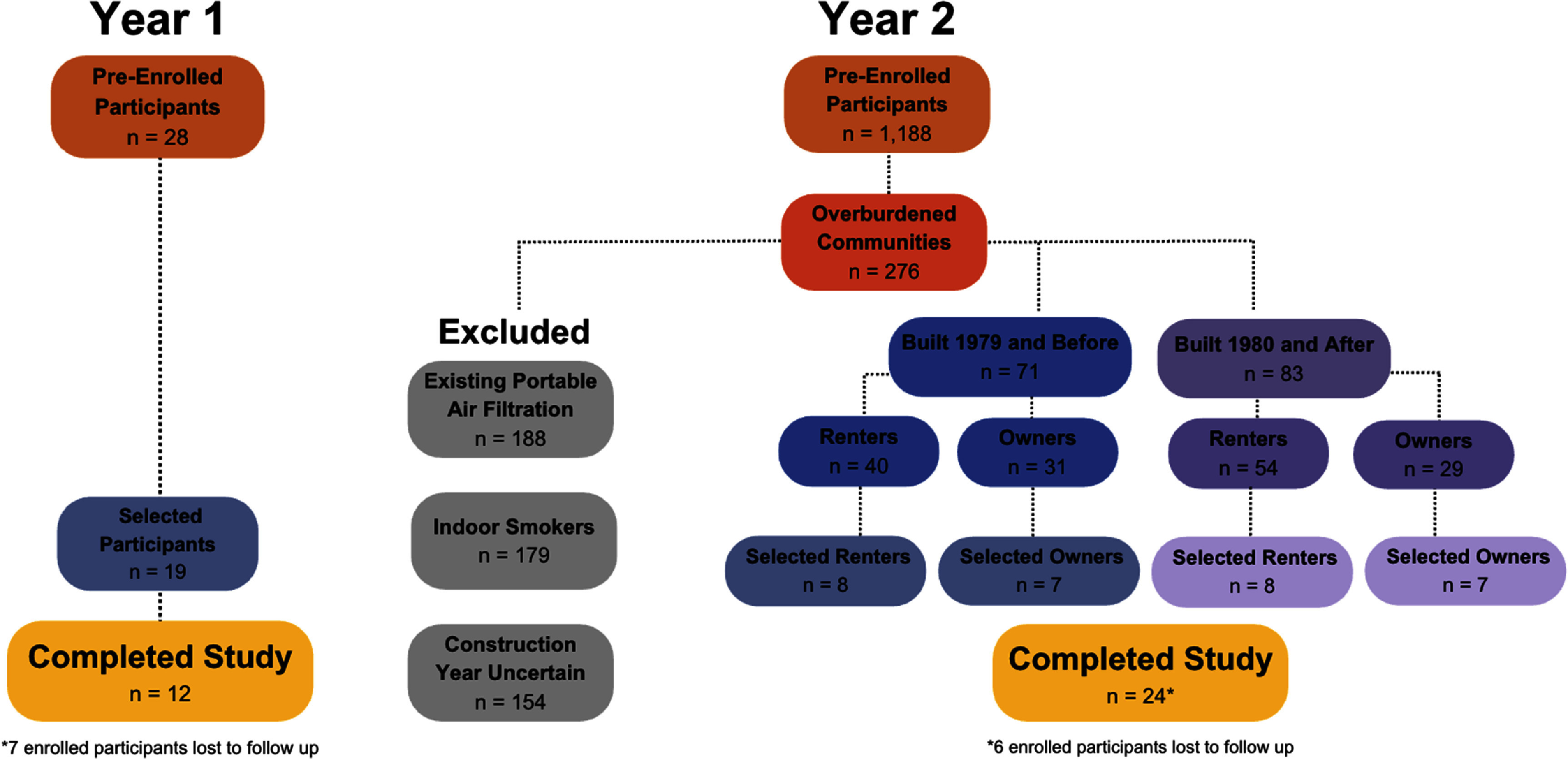
Year 1 (2023) & year 2 (2024) participant criteria.

This pilot study took place over the course of two years, between 2023 and 2024, using iterative methods. Two cohorts of households were recruited, one per study year, from King Count the most populous county in Washington State and the Pacific Northwest region of the US. The year 1 cohort was recruited through outreach and engagement at community health resource fairs and via community-based organization (CBO) listservs. In year 2, a geotargeted social media advertisement was used to solicit interested King County residents. During year 1, households were deemed eligible to participate if they had a secure Wi-Fi connection and a consenting adult proficient in English. Priority was given to households located in South King County, an area disproportionately impacted by outdoor air pollution and with fewer regulatory air quality sensors according to the Washington State Department of Health’s Environmental Health Disparities Map [45]. In year 2, eligibility requirements remained the same as year 1, with additional exclusion factors and enrollment limited to priority zip codes (figure 1). These priority zip codes were defined within ‘overburdened communities highly impacted by air pollution’ as identified by the Washington State Department of Ecology [46]. Given the much greater interest solicited with year 2 recruitment methods versus those of year 1, and due to project budgetary constraints, a random number generator was used to select 30 households within the priority zip codes, across a range of year-built and renter/owner categories. In both years, initial consent was obtained from participants through a pre-enrollment survey and subsequently confirmed in an enrollment survey. The enrollment survey preamble described the study objectives and activities, and it confirmed that participants’ data would be de-identified if used in future research. Participants consented to continued participation in the study by confirming their agreement to the study conditions. In year 1, participants were enrolled on a rolling basis, with participants entering the study from 17th May–1st June 2023. In year 2, all participants began the study on July 8th, 2024. The study was approved by the University of Washington Institutional Review Board.

**Figure 2.**
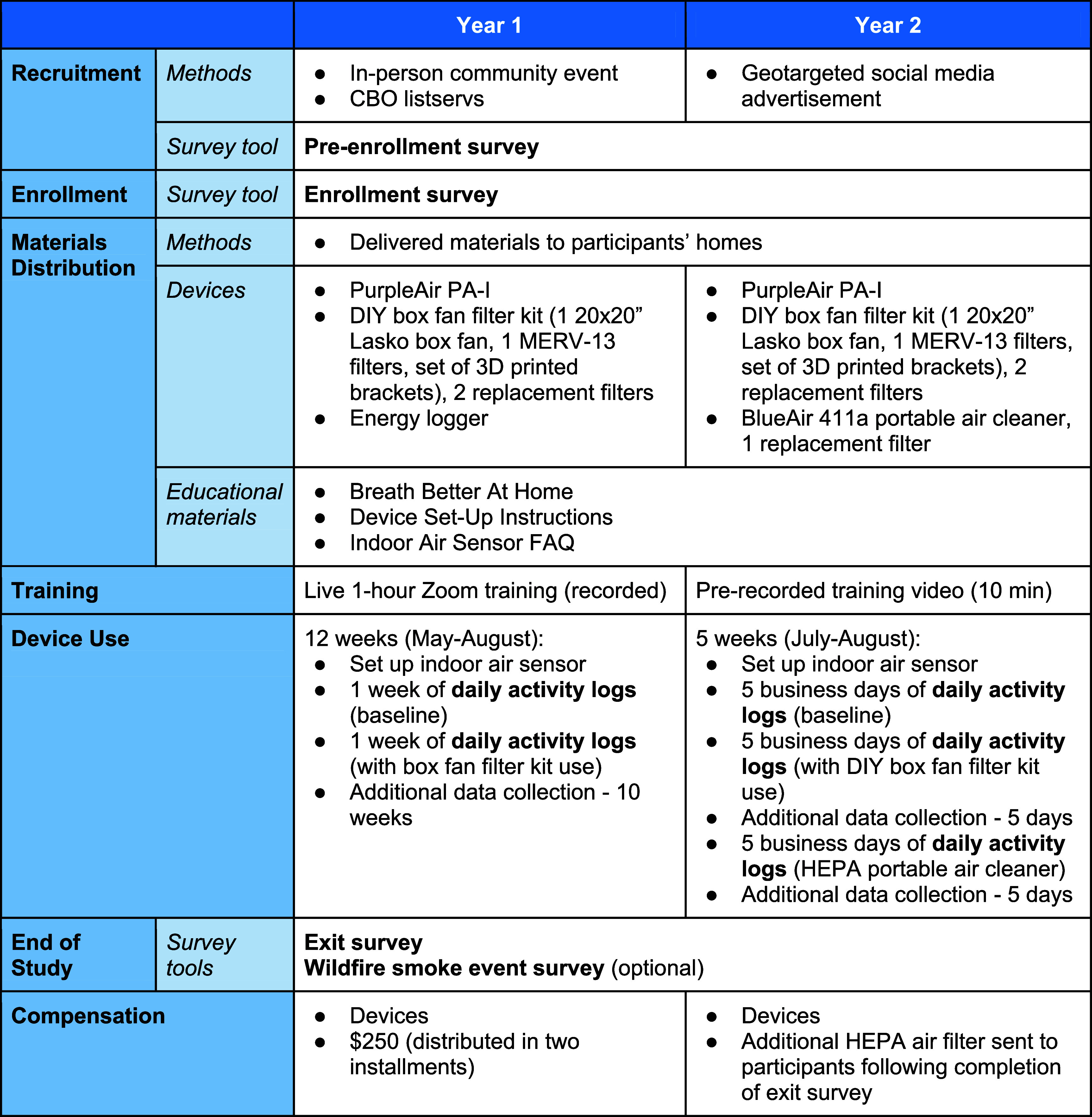
Study procedures, years 1 and 2.

Across both cohorts, participating households were provided with a low-cost air sensor (PurpleAir PA-I) and a DIY box fan filter kit (one 20″x20″x1″ Lasko box fan, three MERV-13 filters, and a set of 3D printed brackets to attach the filter to the fan) (figure 2). In year 1, participants received an energy logger connected to the box fan to record when the device was in use. In year 2, participants also received a dedicated HEPA portable air cleaner (BlueAir 411a) with a clean air delivery rate similar to that of the DIY box fan filter kit for performance comparison.

Prior to device setup and use, participants reviewed educational materials describing the study procedure, device operating instructions, and common household activities and behavior that can contribute to poor IAQ. In year 1, participants were compensated for their time spent participating in the study and were permitted to keep their devices. In year 2, participants were permitted to keep their devices, including the BlueAir 411a, the value of which was comparable to the year 1 compensation.

In year 1, members of the research team delivered study materials to participants. The research team hosted a 1 h virtual training on device setup and study procedures, which was recorded for participants unable to attend live. Members of the research team were available to troubleshoot device setup with study participants and primarily assisted with connecting air sensors to Wi-Fi networks. In year 2, the research team delivered study materials to participants in two installments, the first delivery consisted of the educational materials, low-cost air sensor, and DIY box fan filter kit and the second consisted of the HEPA portable air cleaner and a replacement filter. In year 2, the research team also produced a short instructional video describing the setup of each of the three devices. Research team members were available via email to address participants’ questions or concerns throughout the duration of the study.

### 2.2. Survey administration

Across both years, study participants completed the same series of surveys, though questionnaires were amended slightly from year 1 to year 2. Interested parties first completed a pre-enrollment survey. Those who met the inclusion criteria were invited to complete an enrollment survey. In year 1, following device distribution and air sensor setup, study participants completed a daily activity log for a period of one week (5 business days) to establish baseline air quality data where no additional air filtration methods were applied. After one week, they were instructed to set up their DIY box fan filter kit and complete another week of activity logs. Participants were instructed to use their DIY box fan filters when they noticed poor IAQ, as indicated by a color change on their PurpleAir sensors. In year 2, participants also completed one week (5 business days) of daily activity logs where no air filtration devices were used to establish a baseline. Next, participants completed one week of activity logs while only using the DIY box fan filter kit, with the following week serving as a grace period to finish any uncompleted activity logs. Then participants completed one week of activity logs while only using the HEPA portable air cleaner, with the following week again serving as a grace period to finish any uncompleted activity logs. Again, participants were instructed to use their DIY box fan filters or HEPA portable air cleaners when their PurpleAir sensors indicated poor IAQ. Finally, participants completed an exit survey at the end of the study period, after 12 weeks (year 1) or five weeks (year 2).

Using a mixture of multiple choice and open-ended response, the pre-enrollment survey asked respondents basic questions about their household, including zip code, type of residence (e.g. single family home or apartment), renter or ownership status, and whether members of their household currently use any type of portable air cleaner. Questions about building characteristics were included in this survey to allow the research team to select a representative sample of study participants based on factors including residence location, type of building, and age of building. However, only enough participant interest was solicited during year 2 to apply this information in participant selection.

The enrollment survey was used to formally enroll participants in the study; it asked participants additional questions about the building characteristics of their home (e.g. type of household heating system), their risk perception of indoor air pollution and WFS exposure, and where they access information about air quality. Response options included a mixture of multiple choice, Likert scale, and open-ended response.

The daily activity log asked participants to record observations made about their sensor via multiple choice and open-ended response questions. This survey was designed to collect observations made over a 24 h period and were sent to participants daily for two weeks in year 1, and daily for three one-week increments in year 2. This survey also asked participants to record activities in their home that may have influenced the air quality (e.g. cooking, cleaning, burning candles), if they took action to improve the air quality after observing the sensor change color, and how long their sensors took to return to normal or baseline colors.

All surveys were distributed via email in year 1 and via email or text message in year 2, depending on participant preference. These results are presented in an unpublished article (McKinley *et al*, in progress).

### 2.3. PurpleAir data and analysis

For each participating household, three outdoor proxy sensors were identified by nearest location on the PurpleAir real-time interactive map [47]. Following the completion of each study period, hourly averaged PM_2.5_, temperature, and relative humidity measurements were downloaded from the PurpleAir API for each indoor sensor and every identified outdoor proxy sensor. While indoor PurpleAir sensors have a singular laser counter for detecting particulates (hereafter, channel A), outdoor sensors contain two counters (hereafter, channel A and B) [48]. Raw PM_2.5_ concentrations from sensor channels A (indoor and outdoor sensors) as well as B (outdoor sensors only) were downloaded with the ‘pm2.5_cf_1’ field specified, as recommended by US EPA air quality monitoring program staff. Data were downloaded from the time of enrollment through each summer’s most significant WFS event—21–28th August, 2023 (year 1) and 5–8th September, 2024 (year 2), with a few buffer days following the WFS event. A WFS event was defined by the presence of an active fire, as confirmed by the US EPA’s AirNow Fire & Smoke Map [49], and a sustained AQI of moderate (51–100) for a 24 h period or longer, or if the AQI jumped to the unhealthy for sensitive groups category (101–150) at any point within King County.

All analyses of the PurpleAir data were performed using R statistical software (v2024.12.1 + 563). Data were initially visualized with histograms, scatterplots, density plots, and time series graphs to verify trends and identify outliers. The proportion of missing hourly observations across study years and during WFS events versus ambient periods was calculated. Seven outdoor sensors were removed for their extremely low, extremely high, or missing PM_2.5_, temperature, and/or humidity measurements throughout the entirety of the study period (e.g. sensors with zero PM_2.5_ observations or negative average temperature observations), as such values indicate sensor malfunctions. Outdoor sensor data were then cleaned in accordance with US EPA guidance [50]. Specifically, outdoor data were excluded if both the absolute difference between the channel A and B hourly averages was equal to or greater than 5 *μ*g m^−3^ and the percent difference between the channel A and B hourly averages was equal to or greater than 70% [50]. Hourly observations for channels A and B were averaged to yield a single PM_2.5_ *μ*g m^−3^ hourly estimate. Finally, these hourly estimates were averaged across each group of outdoor proxy sensors and paired with their corresponding indoor hourly PM_2.5_ estimate. Since indoor PurpleAir sensors only have a single channel, the steps for the outdoor sensors data quality control could not be applied to the indoor sensor data. Next, the updated US EPA correction factor for PurpleAir data was applied to every indoor and outdoor observation [51]. This correction factor adjusts each observation given the corresponding relative humidity measurement and separately considers extreme (>343 *μ*g m^−3^) values.

Summary statistics of indoor and outdoor PM_2.5_ concentrations across both study years were generated. Each participant’s daily average time series indoor and outdoor PM_2.5_ data were plotted for each study year, and average trend lines were calculated. As the PM_2.5_ data were right skewed, PM_2.5_ concentrations were log-transformed for the subsequent modeling analyses. To evaluate the efficacy of air filtration use, a linear mixed-effects model was used with indoor PM_2.5_ as the outcome, filtration type (i.e. baseline, DIY box fan filter kit, or HEPA portable air cleaner) as a fixed effect, and a random intercept for each household. This analysis was only performed on the year 2 cohort, as the year 1 cohort did not use the HEPA portable air cleaner. Since the exact timing of air filtration use could not be verified, the air filtration use period was defined as the week in which participants were instructed to use the respective devices when they noticed poor IAQ. Furthermore, given that filtration interventions were implemented in a fixed temporal sequence (DIY box fan filter followed by the HEPA portable air cleaner), the potential for confounding by time must be noted. Analyses relied on within-household comparisons over relatively short periods that did not coincide with WFS events, helping to minimize temporal bias. Nonetheless, to better account for the temporal variation in conditions across the periods of air filtration use, a sensitivity analysis was performed with the air filtration efficacy model adjusted for outdoor PM_2.5_ concentrations.

Additionally, to assess the association between WFS and building characteristics, a linear mixed-effects regression model was utilized. Specifically, fixed effects were included for the occurrence of a WFS event, outdoor temperature, exact year built (data obtained from the King County Parcel Assessor) [52], stove type, household heating type (i.e. heating that circulates air versus those that do not circulate air), air conditioner presence, appraised value per $100 000 (data obtained from the King County Parcel Assessor) [52], study year (i.e. year 1 versus year 2), and residence status (i.e. rent versus own). Random effects were also included to allow each household to have its own intercept (i.e. baseline indoor PM_2.5_) and its own slope for a WFS event (i.e. different smoke responses across households). The model is as follows:

${\mathrm{log}}\left( {{Y_{ij}}} \right) = {\beta _0} + {\beta _1}{\mathrm{smok}}{{\mathrm{e}}_{ij}} + X_{ij}^ \top \beta + {u_{0i}} + {u_{1i}}{\mathrm{smok}}{{\mathrm{e}}_{ij}} + { \in _{ij}}$, where:

${Y_{ij}}$: indoor PM_2.5_ measurement for household *i* at time *j*

${\mathrm{smok}}{{\mathrm{e}}_{ij}}$: indicator for WFS event

${X_{ij}}$: vector of fixed-effect covariates

${u_{0i}}{\text{ }}$: random intercept for household *i*

${u_{1i}}$: random slope for the effect of smoke in household *i*

${ \in _{ij}}$: residual error

For linear mixed-effects models, 95% confidence intervals were calculated using Wald-type intervals based on model-based standard errors. Marginal and conditional *R*^2^ metrics were calculated to evaluate model performance. *T*-values were used to determine significance, with *t* ⩽ −2 and *t* ⩾ 2 considered significant.

## 3. Results

### 3.1. Participant demographics

Our analysis included 12 households in year 1 and 24 households in year 2 for a total of 36 unique households across the full two-year pilot study period, after filtering for participants with insufficient survey participation. Table S1 presents the various participant demographics by each study year and both years combined.

With regards to participant race and ethnicity, most participants across the entire study period self-reported as white (63.89%), Asian (27.78%), and Latinx/Hispanic (19.44%). The remaining participants were Black/African American (8.33%), Middle Eastern (8.33%), and American Indian/Alaska Native (2.78%), with no other races or ethnicities reported. The median number of total occupants per household was 3 occupants. On average, households had more adults aged 18–64 years (1.94 per household) than children aged <18 years (0.97 per household) and older adults aged over 65 (0.28 per household). Less than half (44.44%) of participants reported that at least one person in their household had at least one pre-existing health condition. (e.g. asthma, chronic obstructive pulmonary disease, other respiratory diseases, heart disease, circulatory disease, pregnancy, a previous stroke, or long-COVID-19).

### 3.2. Household building characteristics

Most participants resided in single family homes (63.89%), followed by multi-family units (36.11%). About half of participants owned their home (55.56%) and less than half rented their home (44.44%) (table S2). Half of the participants (50.00%) reported that their household was built in the year 1979 or before, with the other half of participants (50.00%) reporting that their household was built in the year 1980 or after. As a result of our year 2 participant selection criteria, most households did not have indoor smokers (94.44%), with just two households from the year 1 study period indicating they smoked indoors (5.56%).

All households had either electric kitchen stoves (69.44%) or gas stoves (30.56%). Home heating types varied, with about half of households having forced air (47.22%). Other heating types indicated included baseboard heating (13.89%), in-floor heating (8.33%), radiator heating (8.33%), heat pumps (11.11%), and wood stove heating only (2.78%). Three households were unsure of their home heating type (8.33%). Seven households reported filters in their air handling system (19.44%), with filters being either pleated (11.11%) or HEPA (8.33%), and 36.11% of households were unsure about filter use or type. Very few households had furnace fans with a ‘recirculate only’ mode (5.56%) and other households knew that their furnace fans did not have this feature (11.11%) or were unsure (38.89%). Most households did not have air conditioning (63.89%). Households with air conditioning had either whole house air conditioning (25.00%) or a portable window unit (8.33%).

Most participants (83.33%) indicated that they did not use a portable air cleaner in their household prior to the study, while 13.89% of households did. Most participants reported placing their PurpleAir sensor in their household’s living room (41.67%) or a bedroom (25.00%) during the study. Some households (16.67%) reported placing their sensor in an open concept room. Only 5.56% of participants placed their sensor in a dining room, kitchen, or office. The median size of these rooms was 210 sq. ft. in year 1 and 325 sq. ft. in year 2. The median appraised household value was $520 000 in year 1 and $582 000 in year 2.

### 3.3. Trends in indoor and outdoor PM_2.5_ concentrations

There were 26 176 total observation hours of indoor and outdoor PurpleAir data in year 1 and 36 551 in year 2 (table 1) Overall, indoor and outdoor PM_2.5_ concentrations were higher in year 1 compared with year 2. The indoor mean PM_2.5_ was 11.54 *µ*g m^−3^ in year 1 and 8.92 *µ*g m^−3^ in year 2. The outdoor mean PM_2.5_ was 11.10 *µ*g m^−3^ in year 1 and 9.36 *µ*g m^−3^ in year 2. Maximum concentrations were substantially lower in year 2 for both indoor (559.89 *µ*g m^−3^ versus 823.95 *µ*g m^−3^) and outdoor measurements (69.05 versus 292.12 *µ*g m^−3^). There was also reduced variability in year 2 for both indoor (standard deviation (SD) of 25.19 *µ*g m^−3^ in year 1 versus 13.75 *µ*g m^−3^ in year 2) and outdoor (SD of 19.50 *µ*g m^−3^ in year 1 versus 6.13 *µ*g m^−3^ in year 2) measurements. These results align with the differences in observed WFS between the two years: year 1 (2023) had two WFS events (5th July and 21–28th August) of greater intensity, while year 2 (2024) had just one WFS event (5–8th September). Analyses of missing data revealed that 11.83% of hourly observations were missing in 2023, and 8.11% of hourly observations were missing in 2024. Furthermore, missingness was higher during WFS events (16.07%) compared to ambient, non-WFS periods (9.07%). Finally, the proportion of time points where indoor PM_2.5_ exceeded outdoor levels (i.e. where the indoor/outdoor (I/O) ratio was greater than 1) was nearly identical across years, with 36.54% in year 1 and 36.79% in year 2, suggesting consistent relative contributions from indoor sources in both study periods (table 2).

**Table 1.** Summary statistics of hourly observations of indoor and outdoor PM_2.5_ (*µ*g m^−3^) across study years.

		Indoor					Outdoor				
Year	Observations	Mean	Median	Minimum	Maximum	SD	Mean	Median	Minimum	Maximum	SD
1	26 176	11.54	5.29	1.29	823.95	25.19	11.10	6.67	0	292.12	19.50
2	36 551	8.92	6.38	1.16	559.89	13.75	9.36	7.88	0.02	69.05	6.13

**Table 2.** Proportion of observations with an indoor/outdoor (I/O) ratio above 1.

Year	Total observations	Observations with I/O ratio >1	Proportion (%)
1	26 176	9565	36.54
2	36 551	13 446	36.79

**Figure 3.**
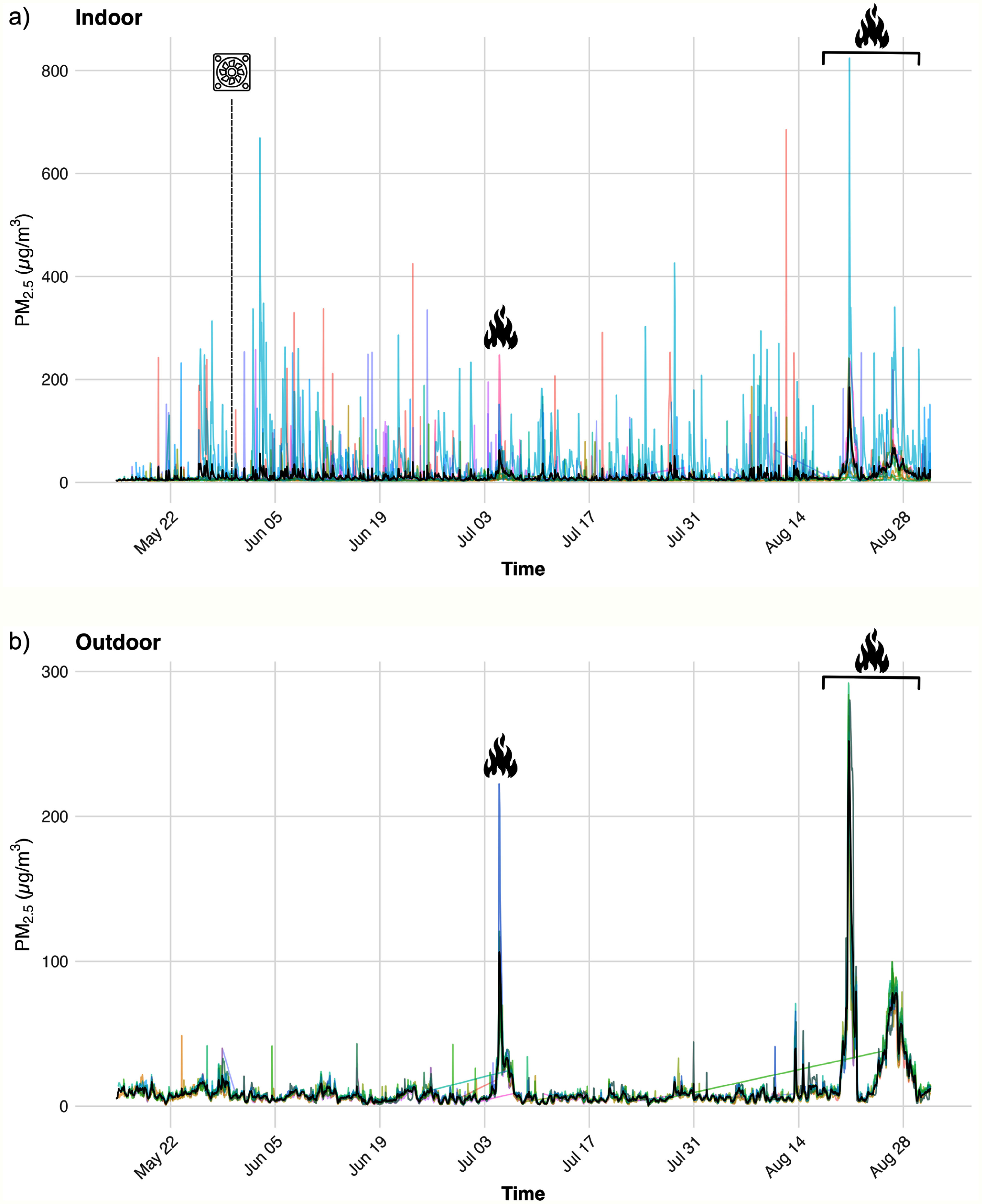
Time series of (a) indoor and (b) outdoor daily PM_2.5_ concentrations during year 1 (2023) of the study. Colors correspond to unique participants. Black line indicates the average concentration. Dashed vertical line and fan icon indicate the first potential start date of DIY box fan filter kit usage, given that a staggered study start was used for year 1 participants. Fire icons and brackets indicate WFS events. Note the different *y*-axis scales. Additionally, note that individual households are not intended to be distinguished; rather, the use of unique colors per household are meant to collectively illustrate the substantial between-and within-household variability.

**Figure 4.**
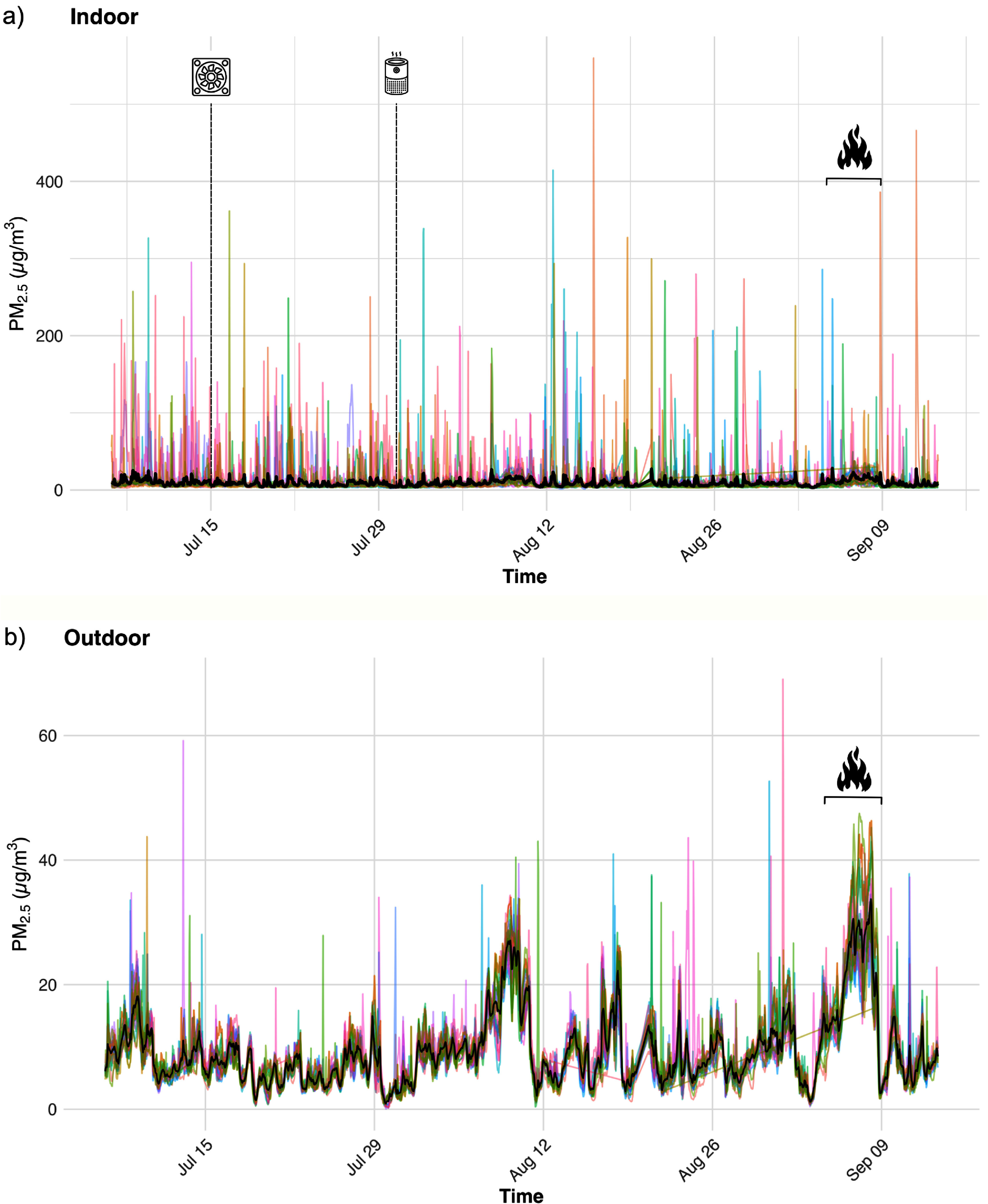
Time series of (a) indoor and (b) outdoor daily PM_2.5_ concentrations during year 2 (2024) of the study. Colors correspond to unique participants. Black line indicates the average concentration. Dashed vertical lines and corresponding icons indicate the starting dates of DIY box fan filter kit and HEPA portable air cleaner usage. Fire icon and bracket indicate the WFS event. Note the different *y*-axis scales. Additionally, note that individual households are not intended to be distinguished; rather, the use of unique colors per household are meant to collectively illustrate the substantial between-and within-household variability.

The time series plots of the observed PM_2.5_ concentrations highlight the much greater variability observed indoors (figures 3(a) and 4(a)) compared to outdoors (figures 3(b) and 4(b)) across both year 1 (figure 3) and year 2 (figure 4). Of note, figure 3(a) includes one household (i.e. blue line) corresponding to a participant who indicated that they smoked indoors. This may explain the large disparity between the indoor observations of this year 1 household and the indoor observations of the other participants. Thus, year 1 indoor data are much more skewed than the corresponding outdoor data in year 1, as well as compared to both the indoor and outdoor data of year 2. This is reflected in the different *y*-axis scales across figures 3 and 4. Resultingly, indoor smoking was added as an exclusion criterion during participant selection for year 2. In the outdoor time series, notable peaks in the average PM_2.5_ concentrations occurred during the WFS events in year 1 (figure 3(b)), as well as during the WFS event in year 2 (figure 4(b)). While not as prominent, the indoor time series data reflected elevated average PM_2.5_ concentrations during these events in both year 1 (figure 3(a)) and year 2 (figure 4(a)).

### 3.4. Air filtration efficacy on indoor PM_2.5_

The results of the linear mixed effects model of air filtration use on indoor PM_2.5_ concentrations (modeled on the log-scale, unadjusted for outdoor PM_2.5_ concentrations) during the dedicated 6 week period in year 2 are shown in table 3. Across all households during the baseline period, the population-average indoor PM_2.5_ concentration was 7.89 *µ*g m^−3^ (95% CI: 7.25, 8.60). During DIY box fan filter kit use, households had, on average, a 21.70% lower indoor PM_2.5_ concentration (95% CI: −23.20%, −20.30%), compared to the baseline period, after accounting for repeated measures within homes. Based on the model estimates, DIY box fan filter kit use corresponded to a population-average geometric mean indoor PM_2.5_ concentration of approximately 6.15 *µ*g m^−3^. In comparison, during HEPA portable air cleaner use, households had, on average, a 4.50% lower indoor PM_2.5_ concentration (95% CI: −6.30%, −2.66%), after accounting for repeated measures within homes. Based on the model estimates, HEPA portable air cleaner use corresponded to a population-average geometric mean indoor PM_2.5_ concentration of approximately 7.57 *µ*g m^−3^. Both air filtration interventions were statistically significant predictors of indoor PM_2.5_ concentrations, with the DIY box fan filter kit showing a much stronger statistical signal (*t* = −25.90) than the HEPA portable air cleaner (*t* = − 4.74). For the random effects, there was moderate between-household variability in baseline indoor PM_2.5_ concentrations, as indicated by the random intercept variance (*σ*^2^ = 0.044). There was substantial within-household temporal variability, as reflected by the residual variance (*σ*^2^ = 0.27). While the air filtration interventions had a statistically significant effect, they explain only a modest proportion (3.60%) of the total variability in indoor PM_2.5_ (marginal *R*^2^ = 0.036). Including between-household differences in the model (i.e. random effects) accounted for a greater proportion of variability (conditional *R*^2^ = 0.17; 17.0%), though a large portion of the variance (83.0%) is unexplained.

**Table 3.** Fixed effects results of air filtration use on log (indoor PM_2.5_).

Term	Estimate	Std. error	*t*-value	95% CI	Exp (estimate)	Exp (95% CI)	% Change
Baseline	2.07	0.044	47.40	(1.98, 2.15)	7.89	(7.25, 8.60)	
DIY air filter	−0.25	0.0094	−25.90	(−0.26, −0.23)	0.78	(0.77, 0.80)	−21.70
HEPA	−0.05	0.0097	−4.74	(−0.065, −0.027)	0.96	(0.94, 0.97)	−4.50

In a sensitivity analysis adjusting for outdoor PM_2.5_, the estimated effects of air filtration were attenuated and differed in magnitude (tables 4 and S3). Adjusting for outdoor PM_2.5_ concentrations, both air filtration interventions were again statistically significant predictors of indoor PM_2.5_. However, lower indoor PM_2.5_ concentrations were observed during the HEPA portable air cleaner use (−12.70% change; 95% CI: −14.19, −11.21) than during the DIY box fan filter use (−7.54% change, 95% CI: −9.09, −5.96), as compared to indoor PM_2.5_ concentrations during the baseline period. This contrasts with the unadjusted model, in which the DIY box fan filter appeared more effective. Additionally, outdoor PM_2.5_ was a strong predictor of indoor PM_2.5_, with each 1 *µ*g m^−3^ increase in outdoor PM_2.5_ associated with a 5.31% increase in indoor concentrations. Model fit improved substantially after adjustment (marginal *R*^2^ = 0.21; conditional *R*^2^ = 0.35), indicating that outdoor air quality explained a large portion of the variability in indoor PM_2.5_.

**Table 4.** Fixed effects results of air filtration use on log (indoor PM_2.5_), adjusted for outdoor PM_2.5_.

Term	Estimate	*t*-value	Exp (estimate)	Exp (95% CI)	% Change
Baseline	1.58	36.20	4.86	(4.46, 5.30)	
DIY air filter	−0.078	−9.05	0.93	(0.91, 0.94)	−7.54
HEPA	−0.14	−15.60	0.87	(0.86, 0.89)	−12.70
Outdoor PM_2.5_	0.052	77.10	1.05	(1.05, 1.05)	5.31

### 3.5. Impact of WFS and building characteristics on indoor PM_2.5_

The fixed effects results of our model of WFS and building characteristics on indoor PM_2.5_ concentrations (modeled on the log-scale) across the two study years are shown in table 5. Our model identified outdoor temperature and WFS events as the strongest predictors of indoor PM_2.5_ concentrations. Specifically, WFS events were associated with a 72.60% (95% CI: 54.50%, 92.90%) increase in indoor PM_2.5_ concentrations as compared to time periods without WFS, and each 1°F increase in outdoor temperature corresponded to a 1.27% (95% CI: 1.22%, 1.31%) increase in indoor PM_2.5_ concentrations, holding all other variables constant. In contrast, household characteristics, including year built, stove type, and heating system, showed minimal impact on IAQ. Households’ appraised value showed a modest but statistically significant effect, with each $100 000 increase in value associated with a 6.58% (95% CI: 0.72%, 12.80%) increase in indoor PM_2.5_ concentrations. Households without air conditioning had 19.50% (95% CI: −6.18%, 52.13%) higher indoor PM_2.5_ concentrations compared to households with air conditioning, though this association was not statistically significant.

**Table 5.** Fixed effects results of building characteristics on log (indoor PM_2.5_).

Term	Estimate	Std. error	*t*-value	95% CI	Exp (estimate)	Exp (95% CI)	% Change
(Intercept)	−2.17	3.01	−0.72	(−8.07, 3.74)	0.12	(0.000 31, 42.10)	
WFS event	0.55	0.057	9.65	(0.44, 0.66)	1.73	(1.55, 1.93)	72.60
Outdoor temperature	0.013	0.000 24	52.40	(0.012, 0.013)	1.01	(1.01, 1.01)	1.27
Year built	0.0013	0.0015	0.87	(−0.0017, 0.0043)	1.00	(1.00, 1.00)	0.13
Gas stove	−0.15	0.12	−1.21	(−0.39, 0.092)	0.86	(0.68, 1.10)	−13.90
Non-circulation heating	−0.13	0.12	−1.13	(−0.36, 0.097)	0.88	(0.70, 1.10)	−12.40
No air conditioning	0.18	0.12	1.44	(−0.064, 0.42)	1.19	(0.94, 1.52)	19.50
Appraised value	0.064	0.029	2.21	(0.0072, 0.12)	1.07	(1.01, 1.13)	6.58
Study year 2	0.15	0.10	1.47	(−0.051, 0.35)	1.16	(0.95, 1.42)	16.30
Rent	0.14	0.12	1.17	(−0.094, 0.37)	1.15	(0.91, 1.45)	14.80

Random effects estimates suggest that households varied not only in their baseline indoor PM_2.5_ levels but also in their sensitivity to WFS infiltration. The random intercept variance indicated that households differed modestly in their underlying indoor PM_2.5_ concentrations (*σ*^2^ = 0.079), while the random slope variance for WFS events showed that the impact of WFS on indoor PM_2.5_ varied across homes (*σ*^2^ = 0.11). Residual variability remained the dominant source of variation, as indicated by the higher residual variance (*σ*^2^ = 0.31). The fixed effects explained 14.0% of the total variation in indoor PM_2.5_ (marginal *R*^2^ = 0.14), while the full mixed model (i.e. including household-level random intercepts and random WFS event slopes) explained 36.0% of the total variance (conditional *R*^2^ = 0.36). This indicates that between-household variability accounted for a substantial portion of the model’s explanatory power.

## 4. Discussion

As wildfire frequency and intensity have increased, the Pacific Northwest has experienced longer WFS seasons and worsened air quality days [7, 53]. With climate change exacerbating these conditions, policies and actions that support household-level interventions and indoor air pollution exposure reduction strategies will increasingly serve as important measures for the protection of public health and long-term risk reduction from the harms of rising levels of ambient outdoor air pollution. This multi-year, participatory science study evaluated IAQ with low-cost, PurpleAir sensors across King County, WA households in 2023 and 2024 to better understand the influence of WFS and building characteristics, as well as the efficacy of different air filtration systems. Across diverse household and building characteristics, we identified WFS events and outdoor temperature as the strongest predictors of elevated indoor PM_2.5_ concentrations, while appraised household value was the only building characteristic that was significantly associated with indoor PM_2.5_ concentrations. In the 2024 cohort, we found differential impacts of DIY box fan filter kit and HEPA portable air cleaner use, though both were significantly associated with lower indoor PM_2.5_ concentrations. These findings have important implications for the use of low-cost air sensors and portable air filters as tools for adapting to the increasing occurrence of WFS, as well as for local-level resilience building.

### 4.1. Demographics and building characteristics

Housing is increasingly being recognized as a social determinant of health, especially in the context of climate change [54, 55]. Sub-standard home environments, including those that allow for outdoor pollutants and contaminants to infiltrate indoors, can adversely impact respiratory health, physical injury, mental health, and the transmission of infectious diseases. With Americans spending 90% of their time, on average, indoors, housing quality represents a significant insulating barrier between individuals and increasingly frequent climate-sensitive hazards, such as extreme heat, extreme cold, heavy rainfall, flooding, and WFS [27]. This suggests that indoor environmental conditions, including potable water quality, temperature, and IAQ, should be closely monitored to provide accurate estimates of individuals’ true exposures to sources of pollution [56]. Housing and demographic characteristics may therefore be strong individual-level predictors of indoor exposures and associated health outcomes. In our study, we sought to robustly characterize these attributes among our participating households. Our study had a greater proportion of American Indian/Alaska Native, Asian, Black/African American, white, and Hispanic and Latinx households compared to the 2020 King County census [57]. While challenging to compare to population-level estimates, a substantial proportion (44%) of participating households in this study reported in the enrollment survey that they had at least one household member with a pre-existing health condition that could be influenced by air pollution. This suggests that individuals with pre-existing conditions, who are perhaps more aware of IAQ and its influence on health than those without pre-existing conditions, may have been more likely to enroll in our study.

Regarding building characteristics, participating households were diverse in their type, age, ownership status, stove type, heating type, air conditioner presence and type, prior portable air cleaner use, and where PurpleAir sensors were installed. While many of our participants’ building characteristics mirrored those of greater King County, our participants notably had lower air conditioner usage. According to US Census Bureau data from the 2023 American Housing Survey, approximately 64% of households in the Seattle-Tacoma-Bellevue metropolitan reporting region had air conditioning [58], while only 33% of our pilot study participants reported air conditioner presence across both study years. Perhaps individuals who could not cool their indoor air were more aware of the importance of IAQ and air filtration and were thus more likely to enroll in our study, as compared to individuals who had air conditioning and may associate its usage with clean IAQ. Nonetheless, diverse representation of these characteristics positioned us well to evaluate their potential influence on IAQ and provide further evidence of housing’s role as a social determinant of health.

### 4.2. Influence of air filtration use on IAQ

Our evaluation of two low-cost air filtration strategies adds to a growing body of research on reducing indoor exposures to WFS, which is a prominent public health challenge as climate change intensifies wildfire seasons across the western US, including the Pacific Northwest [59–61]. We found that DIY box fan filters produced a substantial reduction in indoor PM_2.5_ concentrations, whereas commercially available HEPA portable air cleaners yielded more modest improvements. These differences are consistent with intervention studies showing that device clean air delivery rate [62], filter type [63], and room placement [64] strongly influence performance. The fixed sequence of participants’ use of the air filtration interventions could partially explain the reduced efficacy of the HEPA portable air cleaners compared to that of the DIY box fan filters, as temporal variation in ambient air quality, seasonality, or participant behaviors were not captured in our main model. Indeed, our sensitivity analysis that adjusted for outdoor PM_2.5_ yielded substantially attenuated filtration effect estimates and reversed the relative magnitude of the interventions, indicating that differences in ambient air quality across intervention periods contributed to the observed effects in unadjusted models. Thus, when ambient conditions are held constant, the HEPA portable air cleaners may be more effective than the DIY box fan filters in reducing indoor PM_2.5_ levels. This is supported by previous work conducted in seven Seattle residences during WFS episodes, which identified significant reductions in PM_2.5_ infiltration with HEPA filtration use, with effectiveness ranging from 48% to 78% [36]. This study also found substantial between-household differences in indoor PM_2.5_ levels, noting this variation reflects unique outdoor PM_2.5_ levels, occupant behaviors, and building characteristics over time and across residences [36]. Along with our findings of high between-household variability, this highlights the need for practical guidance tailored to King County households’ lived environments, rather than blanket recommendations, and the value of using low-cost air quality sensors to ensure interventions provide the intended benefits. Although both interventions were statistically significant, the small marginal *R*^2^ suggests that air filtration alone explains only a fraction of indoor PM_2.5_ variability. This finding aligns with current evidence that other factors, such as building leakage [65], ventilation capabilities [66], and occupant behaviors [67] also strongly influence indoor pollution.

Importantly, our results support growing evidence on accessible, low-cost filtration interventions as scalable public health tools that can reduce exposure disparities for households lacking central air systems or high-efficiency filtration [60, 68]. Concurrently, the substantial unexplained within-home variability suggests a need for future studies to fully integrate building characteristics, behavioral factors, and real-time infiltration rates to refine filtration recommendations during WFS events. Together, these findings reinforce the importance of household-centered strategies for maintaining healthy indoor air and adapting to climate change as WFS becomes a consistent part of life in the Pacific Northwest and beyond.

### 4.3. Influence of WFS and building characteristics on IAQ

Our findings indicate that WFS and outdoor temperature were the dominant drivers of indoor PM_2.5_ concentrations, supporting existing evidence that smoke events can overwhelm building-level protections. The large increase in indoor PM_2.5_ during WFS periods (73%) is consistent with studies showing that tightly sealed homes may still experience substantial smoke infiltration during WFS episodes, particularly when temperatures are high and occupants may increase window opening or mechanical ventilation behaviors [69, 70]. Our identification of the significant role of WFS on indoor PM_2.5_ concentrations with PurpleAir sensors is consistent with previous work that identified higher PM_2.5_ infiltration during a WFS event in a Vancouver healthcare facility using a different brand of low-cost sensors [35]. Notably, a greater proportion of missing data occurred during WFS episodes compared to non-WFS periods. This difference may be due to sensor malfunctions during high PM_2.5_ concentration events, power or internet disruptions during WFS episodes, or participants’ behavioral changes during WFS events. The differential data missingness across the two periods suggests that missing data are not completely at random and may be related to the WFS exposure conditions. Thus, with underrepresentation in the data of the highest exposure periods, our estimates of WFS effects on IAQ may be biased towards the null. Additionally, although household appraised value had a small but statistically significant association with higher indoor PM_2.5_, this effect was modest relative to the influence of outdoor smoke and temperature. Interestingly, higher household appraised value—a proxy for socioeconomic status—was associated with higher indoor PM_2.5_ concentrations. While worse IAQ has typically been documented in lower-income households [71], high-income households in King County may be more likely than low-income households to use gas for their energy needs, as it is more expensive than electricity [72]. Households that rely on gas for heating and cooking typically have worse IAQ due to the greater emission of NO_2_, carbon monoxide, and other combustion-derived pollutants [73]. This could explain our finding if participating households with higher appraised value did more heavily rely on gas-powered energy, though the null result of gas stove influence does not necessarily support this, and other occupant behavioral factors may be involved. Finally, the wide uncertainty for air conditioning illustrates the challenges of detecting the impacts of behavioral and building-level mitigation in real-world settings where air conditioning use varies by outdoor temperature and household preferences.

The random effects further highlight notable differences between households. While some households had higher baseline indoor PM_2.5_, others showed much stronger or weaker WFS infiltration responses. These unique responses are supported by research on differences in building tightness, filtration use, and occupant behaviors across households during smoke events [69]. However, a large portion of the model’s variance remained unexplained, suggesting household behaviors that are difficult to predict (e.g. opening windows, using air conditioning, and using air filtration) may play substantial roles. Overall, the modest goodness of fit metrics (i.e. *R*^2^’s) highlight challenges in predicting indoor PM_2.5_ during WFS events. These findings call for future studies to incorporate comprehensive data on building characteristics and occupant behaviors to better model exposure and inform targeted public health interventions.

### 4.4. Low-cost sensors as a climate adaptation

Collectively, our study highlights the value of leveraging low-cost sensors as a climate adaptation strategy, particularly as WFS events become more frequent and severe in the Pacific Northwest and the western US. Despite requiring thorough data cleaning and quality control checks, low-cost sensors, such as the PurpleAir sensors, can capture fine-scale temporal and spatial variations in indoor PM_2.5_. This underscores their utility in real-world settings where regulatory monitors cannot accurately characterize heterogeneous IAQ conditions. Indeed, a growing body of research shows that low-cost sensor networks can provide timely, household-level information on indoor exposures and smoke infiltration [74, 75], although the evidence on their indoor use is much more limited than on their application outdoors. The substantial amount of between-household variation we observed in our model emphasizes the need for real-time, individualized air quality information that households can use to quickly modify air polluting behaviors or mitigate poor IAQ during periods of both ambient air pollution and WFS events. As wildfire seasons lengthen and WFS events intensify with climate change, widespread use of low-cost indoor sensors can serve as an accessible and scalable approach to improving household preparedness, promoting health literacy, and supporting public health responses.

### 4.5. Strengths

Our study had several notable strengths. First, our multi-year design allowed us to adapt our methods in year 2 following the challenges we experienced in year 1. Specifically, we modified our recruitment method in year 2 to include a social media advertisement, rather than in-person outreach and engagement that was conducted in year 1. This adjustment increased the sample of interested participants by over 42 times and doubled the number of enrolled participants who completed the entirety of the study in year 2. Additionally, given the 37% loss to follow-up in year 1 among the participants who we enrolled and who completed the exit survey, we decided to shorten the study period and length of data collection in year 2. While we had a smaller proportion of loss to follow-up in year 2, it only accounted for 20% of enrolled participants.

Our extensive series of surveys allowed us to collect detailed information on households’ demographics, building characteristics, risk perception of indoor air pollution, activities related to sensors’ color changing, and subsequent actions taken to mitigate poor IAQ. To permit comparability between the years, we made only slight modifications to the survey tools and methods in year 2, notably asking about the volume of the room where the sensor was installed, and obtaining exact year built and appraised household value from the King County Parcel Viewer. We retroactively searched the King County Parcel Viewer for year 1 households to obtain the same variables. Finally, our continuous monitoring of household IAQ with PurpleAir sensors allowed us to document hourly averaged PM_2.5_ concentrations throughout both study years. With these temporally resolved data, we were able to compare within- and between-sensor variability and evaluate the influence of WFS events, building characteristics, and air filtration use (year 2 only) on IAQ.

### 4.6. Limitations

Our study has limitations related to its generalizability, modeling assumptions, and sample size. Prominently, our sample size was low across both study years. This study evolved out of a descriptive pilot study and was never intended to fully characterize IAQ across all King County, WA households. We recruited participants from community health resource fairs, CBO listservs, and online advertising, with participants being allowed to keep the air sensors and filtration devices after the study’s completion. This sampling method and compensation structure may have induced selection bias, as participants who were already aware of IAQ and concerned about WFS exposures may have been more likely to participate and place value on such devices that provide exposure information and health protection. Thus, our findings are not generalizable to other geographic contexts and may not be generalizable to the greater King County area. Larger-scale investigations that include large sample sizes of different household demographics and building characteristics are needed to confirm our findings.

Additionally, we had to make a few assumptions during our modeling of the PurpleAir data. First, we co-identified outdoor proxy sensors from the PurpleAir real-time interactive map that were closest in proximity to our participants’ indoor sensors. This prevented us from knowing how long these sensors had been operating in outdoor conditions, which limited our ability to quality assure and control these outdoor proxies. Indeed, we had to fully remove 7 of these sensors due to substantial missing data or poor correlation between sensor channels. Given the available data, we assumed that the co-identified sensors were the best proxies for outdoor air quality corresponding to our indoor sensors at any given timepoint. We identified a higher proportion of data missingness during WFS events compared to ambient, non-WFS periods. This suggests that our finding of a 73% increase in indoor PM_2.5_ during WFS periods is conservative and likely an underestimation of the true effect of WFS on IAQ as measured by PM_2.5_ concentrations. Additionally, we had to assume that participants used their air filtration devices exactly as instructed during the designated use periods in year 2 (i.e. no filtration use during the baseline period, followed by exclusive use of the DIY box fan filter kit, followed by exclusive use of the HEPA portable air cleaner). In year 1, we distributed energy loggers with the DIY box fan filter kit to capture timestamps of when exactly the device was used. This would have enabled us to exactly specify air filtration use and corresponding IAQ from the timestamped PurpleAir data. However, many energy loggers were improperly installed, and most were not returned at the end of the study. Given their high expense ($309 per unit), we decided to not administer energy loggers in year 2. Thus, in defining the air filtration use periods, we are relying on participants’ commitments to adhere to study procedures, which they consented to during the enrollment period. Future participatory science studies evaluating the efficacy of air filtration devices should be aware of the challenges of documenting device use and develop explicit installation, return, and troubleshooting procedures for energy loggers.

While the assumption of participants’ air filtration uses as instructed is fair, it may have been less realistic that participants consistently used the DIY box fan filter kit during the designated time, as participants noted concerns with its noise, limited cooling ability, and potential for overheating (results to be reported in a subsequent publication). In contrast, the HEPA portable air cleaner could be set to an automatic mode, in which it would accelerate air filtration when it detected poor IAQ. During ambient periods, it was mostly quiet and unnoticeable. Thus, the assumption of consistent HEPA portable air cleaner use during the designated period may be more realistic than that of the DIY air filtration device. Finally, the implementation of air filtration devices in a fixed temporal sequence in year 2 (i.e. DIY box fan filter kit followed by the HEPA portable air cleaner) may have introduced confounding by time in our analysis of air filtration efficacy. Although no major WFS events coincided with the filtration intervention periods, other time-varying factors may have influenced the estimated filtration effects between the two periods. Indeed, in a sensitivity analysis adjusting for outdoor PM_2.5_, filtration effect estimates were attenuated and reversed in magnitude, suggesting that the main model results were partially confounded by temporal variation in ambient air quality. Model explanatory power improved after adjustment of outdoor PM_2.5_ (marginal *R*^2^ of 0.21 versus 0.036), though a substantial portion of variability in indoor PM_2.5_ remained unexplained. As such, these findings should be interpreted with caution.

Additionally, we assumed that appraised household value was a valid proxy for participants’ ability to implement household upgrades to adapt to WFS and reduce infiltration of outdoor pollutants (e.g. retrofitting, weatherizing). We decided to include this variable, as we did not capture such information through our participant surveys. In hindsight, it would have been useful to capture data on household income, date of last retrofit/weatherization, and any other household improvements that participants were aware of. While appraised household value may still have been an appropriate proxy, these data were not obtained from participants, but rather from the King County Parcel Viewer, which may not reflect the most up-to-date market values.

Finally, our small sample size may have limited the power of our statistical analyses. We collected over 60 000 h indoor and outdoor PurpleAir data points, but only a total of 36 participating households completed the duration of the study across both years. This limited the power of analyses involving household-level building characteristics. To avoid creating subgroups with very small cell counts, we collapsed categories when appropriate. For example, we dichotomized the heating type variable by those that circulate air (i.e. forced air and heating pumps) and those that do not (i.e. in-floor, baseboard, radiator, wood stove, and ‘unsure’). We also used a continuous variable for the exact year built from the King County Parcel Viewer, rather than the decade-based categorical year built variable from survey responses. While this reduced the heterogeneity in some predictors, they allowed for more stable and interpretable estimates. Nonetheless, our results should be interpreted with caution and support the need for additional studies with larger household samples in King County and other geographic contexts.

## 5. Conclusion

In summary, this multi-year, participatory science study demonstrated that WFS and outdoor temperature are the dominant drivers of elevated indoor PM_2.5_ in King County households, while most building characteristics had minor and/or mixed influence. DIY box fan filter kits and HEPA portable air cleaners both improved IAQ as measured by reduced indoor PM_2.5_ concentrations, though filtration alone explained only some of the observed variability. These findings emphasize the complex interplay between building characteristics, occupant behaviors, and outdoor air quality during WFS events, as well as during ambient periods. Our study highlights the mounting importance of low-cost indoor sensors as accessible tools for monitoring household-level air quality. Such measures can support real-time behavior adaptations and inform equitable climate resilience strategies as WFS events intensify with climate change. While our study was limited by small sample size and reduced generalizability, our findings offer insights into WFS infiltration differences and the potential for low-cost interventions to mitigate household indoor air exposures. Future studies with larger samples and exhaustive data on occupant behaviors and building characteristics are needed to improve exposure models and inform targeted public health recommendations.

## Data Availability

The data cannot be made publicly available upon publication because they contain sensitive personal information. The data that support the findings of this study are available upon reasonable request from the authors. Supplementary Data available at https://doi.org/10.1088/2515-7620/ae69a6/data1.

## References

[1] Mansoor S, Farooq I, Kachroo M M, Mahmoud A E D, Fawzy M, Popescu S M, Alyemeni M N, Sonne C, Rinklebe J, Ahmad P (2022). Elevation in wildfire frequencies with respect to the climate change. J. Environ. Manage..

[2] Siddik M, Islam M, Zaman A, Hasan M (2021). Current status and correlation of fossil fuels consumption and greenhouse gas emissions. Int. J. Energy Environ. Econ..

[3] Gergel D R, Nijssen B, Abatzoglou J T, Lettenmaier D P, Stumbaugh M R (2017). Effects of climate change on snowpack and fire potential in the western USA. Clim. Change.

[4] Xu R, Yu P, Abramson M J, Johnston F H, Samet J M, Bell M L, Haines A, Ebi K L, Li S, Guo Y (2020). Wildfires, global climate change, and human health. New Engl. J. Med..

[5] Westerling A L, Hidalgo H G, Cayan D R, Swetnam T W (2006). Warming and earlier spring increase western U.S. forest wildfire activity. Science.

[6] Halofsky J E, Peterson D L, Harvey B J (2020). Changing wildfire, changing forests: the effects of climate change on fire regimes and vegetation in the Pacific Northwest, USA. Fire Ecol..

[7] Childs M L, Li J, Wen J, Heft-Neal S, Driscoll A, Wang S, Gould C F, Qiu M, Burney J, Burke M (2022). Daily local-level estimates of ambient wildfire smoke PM_2.5_ for the contiguous US. Environ. Sci. Technol..

[8] Reisen F, Duran S M, Flannigan M, Elliott C, Rideout K (2015). Wildfire smoke and public health risk. Int. J. Wildland Fire.

[9] Wegesser T C, Pinkerton K E, Last J A (2009). California wildfires of 2008: coarse and fine particulate matter toxicity. Environ. Health Perspect..

[10] McDuffie E E (2021). Source sector and fuel contributions to ambient PM_2.5_ and attributable mortality across multiple spatial scales. Nat. Commun..

[11] Krasovich Southworth E (2025). The influence of wildfire smoke on ambient PM_2.5_ chemical species concentrations in the contiguous US. Environ. Sci. Technol..

[12] Aguilera R, Corringham T, Gershunov A, Benmarhnia T (2021). Wildfire smoke impacts respiratory health more than fine particles from other sources: observational evidence from Southern California. Nat. Commun..

[13] Xing Y-F, Xu Y-H, Shi M-H, Lian Y-X (2016). The impact of PM_2.5_ on the human respiratory system. J. Thorac. Dis..

[14] Fongsodsri K, Chamnanchanunt S, Desakorn V, Thanachartwet V, Sahassananda D, Rojnuckarin P, Umemura T (2021). Particulate matter 2.5 and hematological disorders from dust to diseases: a systematic review of available evidence. Front. Med. Lausanne.

[15] Habre R (2014). The effects of PM_2.5_ and its components from indoor and outdoor sources on cough and wheeze symptoms in asthmatic children. J. Expo. Sci. Environ. Epidemiol..

[16] Kloog I, Ridgway B, Koutrakis P, Coull B A, Schwartz J D (2013). Long- and short-term exposure to PM_2.5_ and mortality: using novel exposure models: using novel exposure models. Epidemiology.

[17] Li W, Lin G, Xiao Z, Zhang Y, Li B, Zhou Y, Ma Y, Chai E (2022). A review of respirable fine particulate matter (PM_2.5_)-induced brain damage. Front. Mol. Neurosci..

[18] Christensen G M, Li Z, Liang D, Ebelt S, Gearing M, Levey A I, Lah J J, Wingo A, Wingo T, Hüls A (2024). Association of PM_2.5_ exposure and Alzheimer disease pathology in brain bank donors-effect modification by APOE genotype. Neurology.

[19] Sherris A R (2025). Wildfire-specific fine particulate matter and preterm birth: a US ECHO Cohort analysis. Lancet Planet Health.

[20] Krittanawong C (2023). PM_2.5_ and cardiovascular diseases: state-of-the-Art review. Int. J. Cardiol..

[21] Chen G (2021). Mortality risk attributable to wildfire-related PM_2.5_ pollution: a global time series study in 749 locations. Lancet Planet Health.

[22] Ma Y, Zang E, Liu Y, Wei J, Lu Y, Krumholz H M, Bell M L, Chen K (2024). Long-term exposure to wildland fire smoke PM_2.5_ and mortality in the contiguous United States. Proc. Natl Acad. Sci. USA.

[23] Liu Y, Austin E, Xiang J, Gould T, Larson T, Seto E (2021). Health impact assessment of the 2020 Washington state wildfire smoke episode: excess health burden attributable to increased PM_2.5_ exposures and potential exposure reductions. GeoHealth.

[24] Nassikas N J, Chan E A W, Nolte C G, Roman H A, Micklewhite N, Kinney P L, Carter E J, Fann N L (2022). Modeling future asthma attributable to fine particulate matter (PM_2.5_) in a changing climate: a health impact assessment. Air Qual. Atmos. Health.

[25] Neumann J E, Amend M, Anenberg S, Kinney P L, Sarofim M, Martinich J, Lukens J, Xu J-W, Roman H (2021). Estimating PM_2.5_-related premature mortality and morbidity associated with future wildfire emissions in the western US. Environ. Res. Lett..

[26] Stone S L (2021). Wildfire Smoke: A Guide for Public Health Officials.

[27] US EPA (2017). Indoor air quality. https://www.epa.gov/report-environment/indoor-air-quality.

[28] US EPA (2014). The inside story: a guide to indoor air quality. https://www.epa.gov/indoor-air-quality-iaq/inside-story-guide-indoor-air-quality.

[29] Chan W R, Nazaroff W W, Price P N, Sohn M D, Gadgil A J (2005). Analyzing a database of residential air leakage in the United States. Atmos. Environ..

[30] Hugh Henderson B H (2022). Infiltration Guidance for Buildings at Design Conditions.

[31] Kalamees T, Alev Ü, Pärnalaas M (2017). Air leakage levels in timber frame building envelope joints. Build. Environ..

[32] Department of Energy (n.d.). Whole-house ventilation. https://www.energy.gov/energysaver/whole-house-ventilation.

[33] Ebrahimifakhar A, Poursadegh M, Hu Y, Yuill D P, Luo Y (2024). A systematic review and meta-analysis of field studies of portable air cleaners: performance, user behavior, and by-product emissions. Sci. Total Environ..

[34] Kumar P, Morawska L, Martani C, Biskos G, Neophytou M, Di Sabatino S, Bell M, Norford L, Britter R (2015). The rise of low-cost sensing for managing air pollution in cities. Environ. Int..

[35] Nguyen P D M, Martinussen N, Mallach G, Ebrahimi G, Jones K, Zimmerman N, Henderson S B (2021). Using low-cost sensors to assess fine particulate matter infiltration (PM_2.5_) during a wildfire smoke episode at a large inpatient healthcare facility. Int. J. Environ. Res. Public Health.

[36] Xiang J, Huang C-H, Shirai J, Liu Y, Carmona N, Zuidema C, Austin E, Gould T, Larson T, Seto E (2021). Field measurements of PM_2.5_ infiltration factor and portable air cleaner effectiveness during wildfire episodes in US residences. Sci. Total Environ..

[37] He J, Huang C-H, Yuan N, Austin E, Seto E, Novosselov I (2022). Network of low-cost air quality sensors for monitoring indoor, outdoor, and personal PM_2.5_ exposure in Seattle during the 2020 wildfire season. Atmos. Environ..

[38] Technical Summary A (2018). Residential air cleaners: a technical summary.

[39] Carvlin G (2023). Washington smoke blog. https://wasmoke.blogspot.com/2023/.

[40] Puget Sound Clean Air Agency (2024). Air quality data summary for 2024. https://pscleanair.gov/615/Data-Summary.

[41] Puget Sound Clean Air Agency (2024). 2023 air quality data summary.

[42] Peterson J (2021). Washington smoke blog. https://wasmoke.blogspot.com/2021/.

[43] Batchelor J (2022). Washington smoke blog. https://wasmoke.blogspot.com/2022/.

[44] Dhammapala R (2020). Washington smoke blog. https://wasmoke.blogspot.com/2020/.

[45] Washington Environmental Health Disparities Map (n.d.). Washington state department of health. https://doh.wa.gov/data-and-statistical-reports/washington-tracking-network-wtn/washington-environmental-health-disparities-map.

[46] Ellison R (2023). Improving Air Quality in Overburdened Communities Highly Impacted by Air Pollution.

[47] PurpleAir (n.d.). PurpleAir real-time air quality map. https://map.purpleair.com/air-quality-standards-us-epa-aqi.

[48] PurpleAir Community (2023). What are channel A and channel B?. https://community.purpleair.com/t/what-are-channel-a-and-channel-b/3643.

[49] Washington Department of Ecology (n.d.). Washington Smoke Blog. https://wasmoke.blogspot.com/.

[50] Barkjohn K, Holder A, Frederick S, Clements A (2020). PurpleAir PM_2.5_ U.S. correction and performance during smoke events.

[51] Johnson Barkjohn K, Holder A, Clements A, Fredericke S, Evans R (2021). Sensor data cleaning and correction: application on the airnow fire and smoke map. https://cfpub.epa.gov/si/si_public_record_Report.cfm?LAB=CEMM%2526dirEntryID=353088.

[52] King County, WA (n.d.). King County Parcel Viewer. https://www.kingcounty.gov/en/dept/kcit/data-information-services/gis-center/maps-apps/parcel-viewer.

[53] Buchholz R R (2022). New seasonal pattern of pollution emerges from changing North American wildfires. Nat. Commun..

[54] Bentley R, Mason K, Jacobs D, Blakely T, Howden-Chapman P, Li A, Adamkiewicz G, Reeves A (2025). Housing as a social determinant of health: a contemporary framework. Lancet Public Health.

[55] Li A, Toll M, Chapman R, Howden-Chapman P, Hernández D, Samuelson H, Woodward A, Bentley R (2025). Housing at the intersection of health and climate change. Lancet Public Health.

[56] Kotzias D (2005). Indoor air and human exposure assessment–needs and approaches. Exp. Toxicol. Pathol..

[57] King County (n.d.). Demographics. https://www.kingcounty.gov/en/dept/executive/governance-leadership/performance-strategy-budget/regional-planning/demographics.

[58] US Census Bureau—American Housing Survey (n.d.). 2023 Seattle—heating, air conditioning, and appliances. https://www.census.gov/programs-surveys/ahs/data/interactive/ahstablecreator.html?s_areas=42660%2526s_year=2023%2526s_tablename=TABLE3%2526s_bygroup1=1%2526s_bygroup2=1%2526s_filtergroup1=1%2526s_filtergroup2=1.

[59] Kirk W M (2017). Indoor air quality and wildfire smoke impacts in the Pacific Northwest. Sci. Technol. Built. Environ..

[60] Fisk W J, Chan W R (2017). Health benefits and costs of filtration interventions that reduce indoor exposure to PM_2.5_ during wildfires. Indoor Air.

[61] May N W, Dixon C, Jaffe D A (2021). Impact of wildfire smoke events on indoor air quality and evaluation of a low-cost filtration method. Aerosol. Air Qual. Res..

[62] Küpper M, Asbach C, Schneiderwind U, Finger H, Spiegelhoff D, Schumacher S (2019). Testing of an indoor air cleaner for particulate pollutants under realistic conditions in an office room. Aerosol. Air Qual. Res..

[63] Srikrishna D (2022). Can 10× cheaper, lower-efficiency particulate air filters and box fans complement high-efficiency particulate air (HEPA) purifiers to help control the COVID-19 pandemic?. Sci. Total Environ..

[64] Novoselac A, Siegel J A (2009). Impact of placement of portable air cleaning devices in multizone residential environments. Build. Environ..

[65] Chan W R, Joh J, Sherman M H (2013). Analysis of air leakage measurements of US houses. Energy Build.

[66] Mannan M, Al-Ghamdi S G (2021). Indoor air quality in buildings: a comprehensive review on the factors influencing air pollution in residential and commercial structure. Int. J. Environ. Res. Public Health.

[67] Apte K, Salvi S (2016). Household air pollution and its effects on health. F1000Res.

[68] Huang G, Zhou W, Qian Y, Fisher B (2019). Breathing the same air? Socioeconomic disparities in PM_2.5_ exposure and the potential benefits from air filtration. Sci. Total Environ..

[69] Liang Y, Sengupta D, Campmier M J, Lunderberg D M, Apte J S, Goldstein A H (2021). Wildfire smoke impacts on indoor air quality assessed using crowdsourced data in California. Proc. Natl Acad. Sci. USA.

[70] Shrestha P M, Humphrey J L, Carlton E J, Adgate J L, Barton K E, Root E D, Miller S L (2019). Impact of outdoor air pollution on indoor air quality in low-income homes during wildfire seasons. Int. J. Environ. Res. Public Health.

[71] Ferguson L, Taylor J, Davies M, Shrubsole C, Symonds P, Dimitroulopoulou S (2020). Exposure to indoor air pollution across socio-economic groups in high-income countries: a scoping review of the literature and a modelling methodology. Environ. Int..

[72] Bureau of Labor Statistics (2025). Average energy prices, Seattle-Tacoma-Bellevue—December 2024. https://www.bls.gov/regions/west/news-release/averageenergyprices_seattle.htm.

[73] Vardoulakis S, Giagloglou E, Steinle S, Davis A, Sleeuwenhoek A, Galea K S, Dixon K, Crawford J O (2020). Indoor exposure to selected air pollutants in the home environment: a systematic review. Int. J. Environ. Res. Public Health.

[74] Abraham S, Li X (2014). A cost-effective wireless sensor network system for indoor air quality monitoring applications. Proc. Comput. Sci..

[75] Connolly R E, Yu Q, Wang Z, Chen Y-H, Liu J Z, Collier-Oxandale A, Papapostolou V, Polidori A, Zhu Y (2021). Long-term evaluation of a low-cost air sensor network for monitoring indoor and outdoor air quality at the community scale. Sci. Total Environ..

